# Contact allergy to aluminium induced by commonly used pediatric vaccines

**DOI:** 10.1186/s40169-016-0129-y

**Published:** 2017-01-04

**Authors:** E. Bergfors, B. Trollfors, A. Inerot, A. Gente Lidholm

**Affiliations:** Department of Primary Health Care, Institute of Medicine, Sahlgrenska Academy, University of Gothenburg, Gothenburg, Sweden

We would like to complete the paragraph on Adjuvants (page 6) in the review *Vaccination in children with allergies to non active vaccine components* by Francheschini et al. [1] which was initiated by the Italian Pediatric Society of Allergy and Immunology in 2013 and published in Clinical and Translational Medicine in 2015.

As mentioned in the review, aluminium (Al) salts are widely used as adjuvants in diphtheria-tetanus-pertussis (DTP) and hepatitis A and B vaccines. The list can be completed with pneumococcal and meningococcal conjugate vaccines, which today are included in the national vaccination schedules in most countries in Europe and the Americas, and also in vaccines against human papilloma virus (HPV) and tick-bore encephalitis (TBE).

According to the authors, the most known and frequent reaction to Al salts is “a palpable nodule at the injection site”. This sounds harmless enough—but in typical cases the nodules are most annoying to the child due to severe pruritus for a very long time [2, 3]. Besides, most children with persistent itching vaccination granulomas become sensitized against Al [4].

Itching vaccination granulomas are described since 1960 [5] but considered very rare [6] until the 1990s when they were reported in 745 of 76,000 children participating in studies on a monocomponent acellular pertussis vaccine in Sweden [7]. Since then, another 102 children in Sweden who received commercial DTaP-polio-Hib-(HepB) combinations (Infanrix^®^, Pentavac^®^) and/or pneumococcal vaccines (Prevenar, Synflorix) are described [4, 8, 9]. The vaccines were given intramuscularly in three doses at 3, 5 and 12 months. In a prospective cohort study on 4758 toddlers the frequency of granulomas was 0.63% in those who received a DTaP combination vaccine alone and 1.18% in those who received an Al adsorbed pneumococcal vaccine at the same time. The risk for granulomas increased with the number of Al-containing vaccine doses [4].

The itching nodules appear remarkably late (months or even years) after the vaccination. Histopathological examination shows granuloma formations in which Al crystals can be demonstrated [10]. Clinically, pruritus is the dominating symptom with intense local itching in the vaccination area on the thigh, often causing skin alterations like eczema, hypertrichosis and hyperpigmentation. Intensified itching and swelling of the nodules is often reported when the child has a cold or another infection. After a duration of ½–12 years (median 3–4 years) the nodules eventually disappear and the pruritus ceases.

In some cases nodules were mistaken as tumours leading to unnecessary anxiety, investigations and surgery [11, 12].

Contact allergy to aluminium was verified in 77–95% of children with itching vaccination granulomas by epicutaneous testing with Al Chloride hexahydrate 2% and metallic Al (4, 7, 9). Sensitized individuals have reported contact dermatitis after the use of Al containing deodorants, pharmaceutics (ear drops, antiseptics), sun protectors, tattooing pigments and metallic aluminium [13]. Fortunately, and contrary to earlier belief, the sensitization to aluminium seems to vane with time [14].

The consequences of future vaccination with Al adsorbed vaccines in children who once reacted with itching granulomas and/or contact allergy to Al is only partially studied. Our clinical experience so far is that the risk for new granulomas diminishes with time and is very low when the original one has vanished and the itching ceased. In case of on-going severe pruritus the next dose may be postponed 6–12 months. The Al allergy is a delayed type IV reaction not associated with increased risk for anaphylaxis.

We want to point out that itching granulomas are benign and self-limiting and no cause to refrain from vaccination in consideration of the risk for a serious infectious disease. They are poorly known but easy to recognize once you are aware of them. They should be familiar to all health care staff working with children to avoid mistrust and anxiety in the parents and unnecessary investigations of the child.
